# A molecular analysis of substituted phenylethylamines as potential microtubule targeting agents through in silico methods and in vitro microtubule-polymerization activity

**DOI:** 10.1038/s41598-023-41600-9

**Published:** 2023-09-01

**Authors:** Isadora Rocha De Abreu, Allison Barkdull, James R. Munoz, Robert P. Smith, Travis J. A. Craddock

**Affiliations:** 1 Clinical Systems Biology Group, Institute for Neuro-Immune Medicine, Nova Southeastern University, Fort Lauderdale, FL USA; 2 Department of Psychology and Neuroscience, Nova Southeastern University, Fort Lauderdale, FL USA; 3 Department of Biomedical Engineering, University of Florida, Gainesville, FL USA; 4 Cell Therapy Institute, Dr. Kiran C. Patel College of Osteopathic Medicine, Nova Southeastern University, Fort Lauderdale, FL USA; 5 Departments of Computer Science, and Clinical Immunology, Nova Southeastern University, Fort Lauderdale, FL USA

**Keywords:** Molecular biology, Computational biology and bioinformatics, Virtual drug screening, Neuroscience, Molecular neuroscience

## Abstract

Natural phenethylamines are trace amine neurotransmitters associated with dopamine transmission and related illnesses such Parkinson’s disease, and addiction. Synthetic phenethylamines can have psychoactive and hallucinogenic effects due to their high affinity with the 5-HT_2A_ receptor. Evidence indicates phenethylamines can directly alter the microtubule cytoskeleton being structurally similar to the microtubule destabilizing agent colchicine, however little work has been done on this interaction. As microtubules provide neuron structure, intracellular transport, and influence synaptic plasticity the interaction of phenethylamines with microtubules is important for understanding the potential harms, or potential pharmaceutical use of phenethylamines. We investigated 110 phenethylamines and their interaction with microtubules. Here we performed molecular docking of these compounds at the colchicine binding site and ranked them via binding energy. The top 10% of phenethylamines were further screened based on pharmacokinetic and physicochemical properties derived from SwissADME and LightBBB. Based on these properties 25B-NBF, 25C-NBF, and DMBMPP were tested in in vitro microtubule polymerization assays showing that they alter microtubule polymerization dynamics in a dose dependent manner. As these compounds can rapidly cross the blood brain barrier and directly affect cytoskeletal dynamics, they have the potential to modulate cytoskeletal based neural plasticity. Further investigations into these mechanisms are warranted.

## Introduction

Designer drugs are a collection of various substances designed to imitate the effects of controlled substances without being detected or categorized as illegal^1^. As such, drug-regulatory authorities face a significant challenge to control such drugs, which can endanger public health when used improperly. Some substances that are referred to as designer drugs may have approved medical use in different legal jurisdictions or countries, further complicating the matter^1^. In general, designer drugs can be classified into the categories as traditional drugs of abuse, such as stimulants, sedatives, dissociatives, cannabinoids, and psychedelics. Unlike traditional drugs of abuse, newly emerging designer drugs can avoid detection of routine drug screening, and there is often limited information available about their associated adverse effects. Healthcare workers who treat patients under the influence of these drugs must possess an understanding of their mechanism of action and the related clinical complications. Such knowledge is crucial for ensuring effective medical care.

While there are many chemically unrelated new psychedelic substances that can be classified as designer drugs, a large proportion are structural or functional analogues of tryptamines or phenethylamines. Natural phenethylamines are a class of aromatic amine alkaloids that function as stimulant neurotransmitters^2^. Compared to the other amine neurotransmitters dopamine, serotonin, histamine and norepinephrine they exist only in trace amounts^3^. Synthetic phenethylamines can have psychoactive, hallucinogenic, and sympathomimetic effects^4^ and include the party drugs methamphetamine/METH (*N*-methylamphetamine), MDMA/ecstasy (3,4-methylenedioxy-methamphetamine), and mescaline (3,4,5-trimethoxyphenethylamine) which are illegal in most countries^5^. As the recreational use of phenethylamines has grown^6^, diverse harmful effects have been reported^1^. The acute adverse effects of phenethylamines including agitation, hallucinations, drowsiness, confusion, mydriasis, aggression, hyperthermia, hypertension, and tachycardia, with more severe long-term adverse effects including acute psychosis, seizures, coma, cerebral edema, long-lasting severe neurological impairment, serotonin syndrome, prolonged respiratory failure, renal failure, multi-organ failure, metabolic acidosis, and rhabdomyolysis^1^ depending on dose. While it is believed that the psychedelic effect of the phenethylamines is mediated by activation of the serotonin receptor 5-HT_2A_, the mechanisms of these adverse effects are less understood.

Phenethylamines show a strong affinity for serotonergic receptors, with the highest affinity for the 5-HT_2A_ receptors, however they also interact with other monoaminergic targets including adrenergic, dopaminergic, and histaminergic receptors, monoamine transporters, and monoamine oxidases^1^. Other potential targets include adenosine receptors, aldose reductases, carbonic anhydrases, dipeptidyl peptidases, dopamine β-hydroxylase, galectin-1 receptors, HIV-1 reverse transcriptase receptors, opioid receptors, peroxisome proliferator-activated receptors, sigma receptors, and trace amine-associated receptors^7^. Beyond these targets, phenethylamine compounds, such as mescaline and its derivatives, are also structurally similar to the microtubule-destabilizing agent colchicine^8^ and may exert effects via direct modulation of cytoskeletal dynamics. Like colchicine mescaline binds to the microtubule constituent protein tubulin, inhibits microtubule dependent axonal transport, and inhibits microtubule and mitotic spindle formation^9^. Conversely, 2-phenethylamine shows a concentration dependent stabilization of microtubules and suggests a biochemical basis for neuromodulation via direct effect on tubulin of phenethylamines^10–12^. The effect of phenethylamines on the microtubule cytoskeleton does not appear to have been studied further than this^13^.

Microtubules are polymers composed of αβ-beta tubulin heterodimers that are responsible for many important functions in cells, from protein transport to cell division, and dendrite interactions with synapses of neurons^14^. Microtubules go through dynamic instability phases of sporadic polymerization that cause microtubule growth by assembling increasing the concentration of guanosine triphosphate (GTP)-tubulin molecules. Dynamic instability also involves sporadic depolymerization causing the microtubule to shrink because the GTP-tubulin assembly gets hydrolyzed to release energy and attain stability in a curved state^15^. Microtubules play a key role in cell division, through the formation of mitotic spindles, a structure that relies on the dynamic instability and precise dynamic stages of microtubule growth and shrinkage during mitosis^16^. As microtubules are essential for proper cell function, their dysfunction has been implicated in many nervous system disorders including the neurodegenerative diseases (i.e. Alzheimer’s, Parkinson’s Huntington’s), neuropsychiatric disorders (i.e. schizophrenia, bipolar disorder, major depression), and neurodevelopmental disorders (i.e. autism)^17^. Microtubule-targeting agents that interfere with microtubule dynamics can contribute to the formation of or exacerbate these conditions. Conversely, with proper regulation and study, such agents could be harnessed for the treatment of many diseases such as cancers^18^, neurodegenerative diseases^19^, and neuropsychological disorders^20^.

As such the goal of this study is to investigate the effect of phenethylamines as microtubule-targeting agents that can either stabilize or destabilize tubulin polymerization. Due to the structural similarity between phenethylamines and the known microtubule-targeting agent colchicine, we performed molecular docking with Autodock Vina^21^ of 110 substituted phenethylamines to the colchicine binding site on tubulin. The top 10% of compounds as ranked by binding energy to tubulin were selected for further analysis. Using SwissADME^22^ we measured pan-assay interference compounds (PAINS) alerts, Brenk alerts, analyzed whether the compound is a P-glycoprotein (Pg-p) substrate, and whether the compound is an inhibitor of five enzymes from the cytochromes P450 (CYP) family. We also used LightBBB^23^ to predict the compound's permeability across the blood brain barrier (BBB). These results were used to select three compounds (25B-NBF (4-bromo-N-[(2-fluorophenyl)methyl]-2,5-dimethoxy-benzene-ethanamine), 25C-NBF (4-chloro-N-[(2-fluorophenyl)methyl]-2,5-dimethoxybenzeneethan-amine), and DMBMPP (2-(2,5-dimethoxy-4-bromobenzyl)-6-(2-methoxy-phenyl) piperidine**)**) from the 110 phenethylamines for in vitro microtubule polymerization testing. Overall, our results indicate that these compounds affect microtubule polymerization dynamics in a dose dependent manner.

## Results

### Computational screening

**Figure 1:**
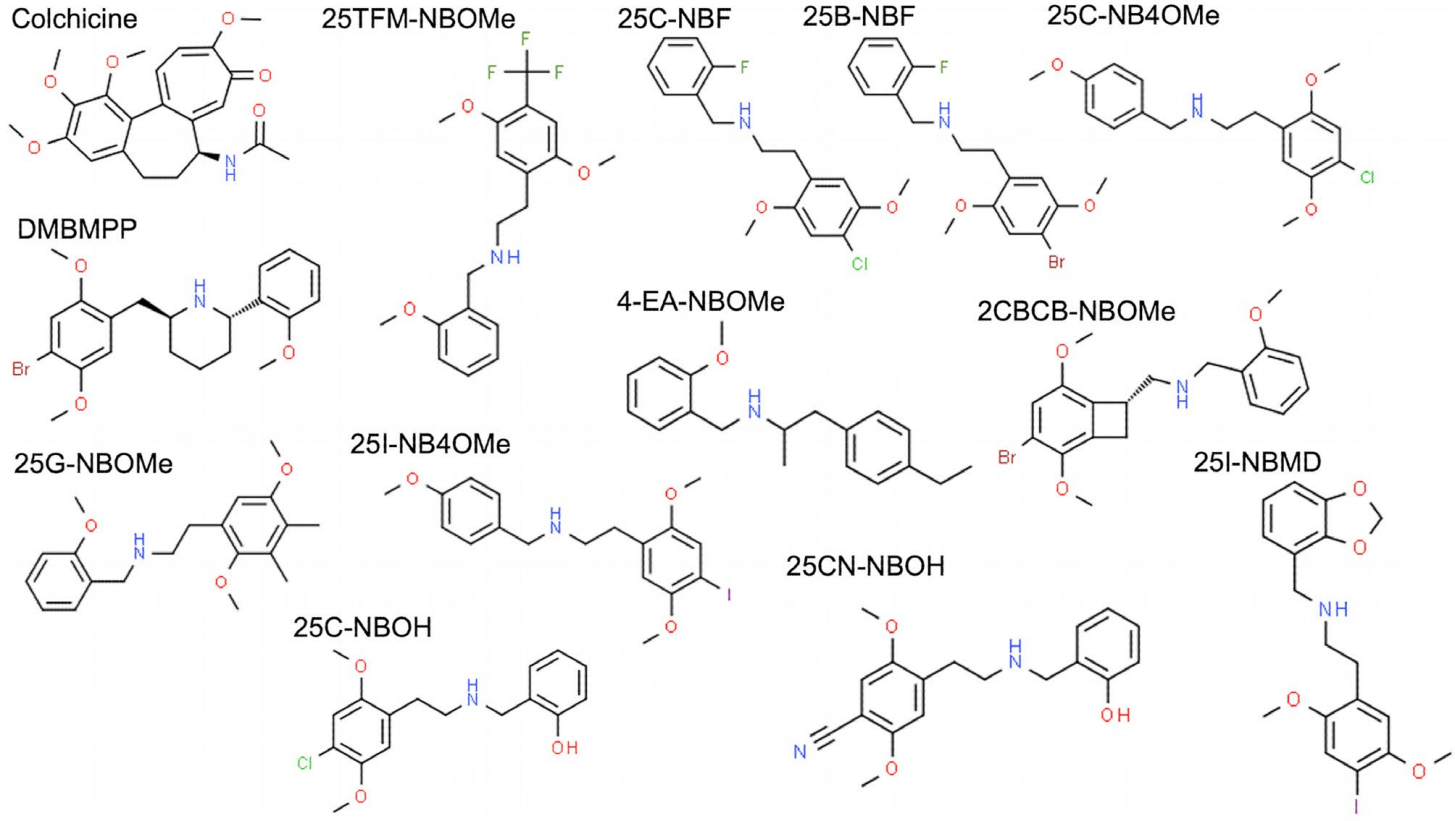
Structure of colchicine and the top 10% of phenethylamines determined by Autodock Vina binding energy to colchicin’es binding site on tubulin.

We used AutoDock Vina^21^ to dock 110 phenethylamine compounds and colchicine to the colchicine binding site on tubulin. The top 10% of the phenethylamines as determined by the strongest binding energy are pictured in Fig. 1 and listed in Table 1 with their binding energies (see Table S1 in Supplementary Information for the complete list). The binding energies of the 110 phenethylamines ranged from − 9 kcal/mol and − 5.5 kcal/mol. The binding energies of the top 10% are comparable to the predicted colchicine binding energy of − 10.8 kcal/mol.

**Table 1:** Top 10% of phenethylamines by binding energy to colchicine’s binding site on tubulin with blood–brain barrier permeability, Pg-p substrate, PAINS alerts, Brenk alerts and interaction with CYP inhibitors.

Molecule	Binding Energy (kcal/mol)*	Cross BBB	Pg-p substrate	PAINS	Brenk	CYP1A2 inhibitor	CYP2C19 inhibitor	CYP2C9 inhibitor	CYP2D6 inhibitor	CYP3A4 inhibitor
Colchicine	− 10.8	No	Yes	0	0	No	No	No	Yes	Yes
DMBMPP	− **9.0**	**Yes**	**No**	**0**	**0**	**No**	**No**	**No**	**Yes**	**Yes**
25TFM-NBOMe	− 8.3	Yes	No	0	0	Yes	Yes	No	Yes	No
25G-NBOMe	− 8.2	Yes	No	0	0	Yes	No	No	Yes	Yes
25C-NBF	− **8.1**	**Yes**	**No**	**0**	**0**	**No**	**Yes**	**No**	**Yes**	**No**
4-EA-NBOMe	− 8.1	Yes	Yes	0	0	Yes	No	No	Yes	Yes
25B-NBF	− **8.0**	**Yes**	**No**	**0**	**0**	**No**	**Yes**	**No**	**Yes**	**No**
25C-NB4OMe	− 8.0	Yes	No	0	0	Yes	Yes	Yes	Yes	Yes
2CBCB-NBOMe	− 8.0	Yes	Yes	0	0	No	No	No	Yes	Yes
25C-NBOH	− 7.9	Yes	No	1	0	Yes	Yes	Yes	Yes	Yes
25CN-NBOH	− 7.9	Yes	No	1	0	Yes	No	No	Yes	Yes
25I-NB4OMe	− 7.9	Yes	No	0	1	Yes	Yes	Yes	Yes	Yes
25I-NBMD	− 7.9	Yes	No	0	1	Yes	Yes	Yes	Yes	Yes

*Lowest energy (LE).

Bold indicates compounds chosen for in vitro polymerization studies.

Assessing the pharmacokinetic properties of the phenethylamines in Table 1 using LightBBB^23^ showed that expectedly, unlike colchicine, the phenylethylamines were able to cross the BBB. From the SwissADME^22^ analysis compounds 25C-NBOH and 25CN-NBOH had PAINS alerts indicating that these compounds were likely to be promiscuous, with multiple binding sites, and to be false positives for pharmacological and biological activity in multiple assays irrespective of their protein target. 25I-NB4OMe and 25I-NBMD were found to have Brenk alerts as identified by 105 chemical fragments that are associated with poor pharmacokinetics, toxicity, chemical reactivity, and metabolic instability^24^ (Table 1). As such, these four compounds were ruled out for further analysis. Compounds 4-EA-NBOMe, and 2CBCB-NBOMe were identified as Pg-p substrates indicating that they are likely readily pumped out of the cell decreasing their ability to exert intracellular effects in the absence of a Pg-p inhibitor^24^ (Table 1). As such, these two compounds were ruled out for further analysis. SwissADME analysis also revealed that all of the top 10% of phenethylamines were found to be inhibitors of at least two of the five major isoforms of cytochrome P450 (CYP1A2, CYP2C19, CYP2C9, CYP2D6, CYP3A4). As this analysis is based on the datasets of Veith et al.^22,25^ inhibiting compounds could be acting as a *bona fide* inhibitor or as a substrate because both will compete for free CYP enzymes. As such, those compounds with the lowest interaction with the CYP enzymes (25B-NBF, 25C-NBF, and DMBMPP) were chosen for further analysis and evaluation via in vitro microtubule polymerization dynamics.

### Binding poses analysis

**Figure 2:**
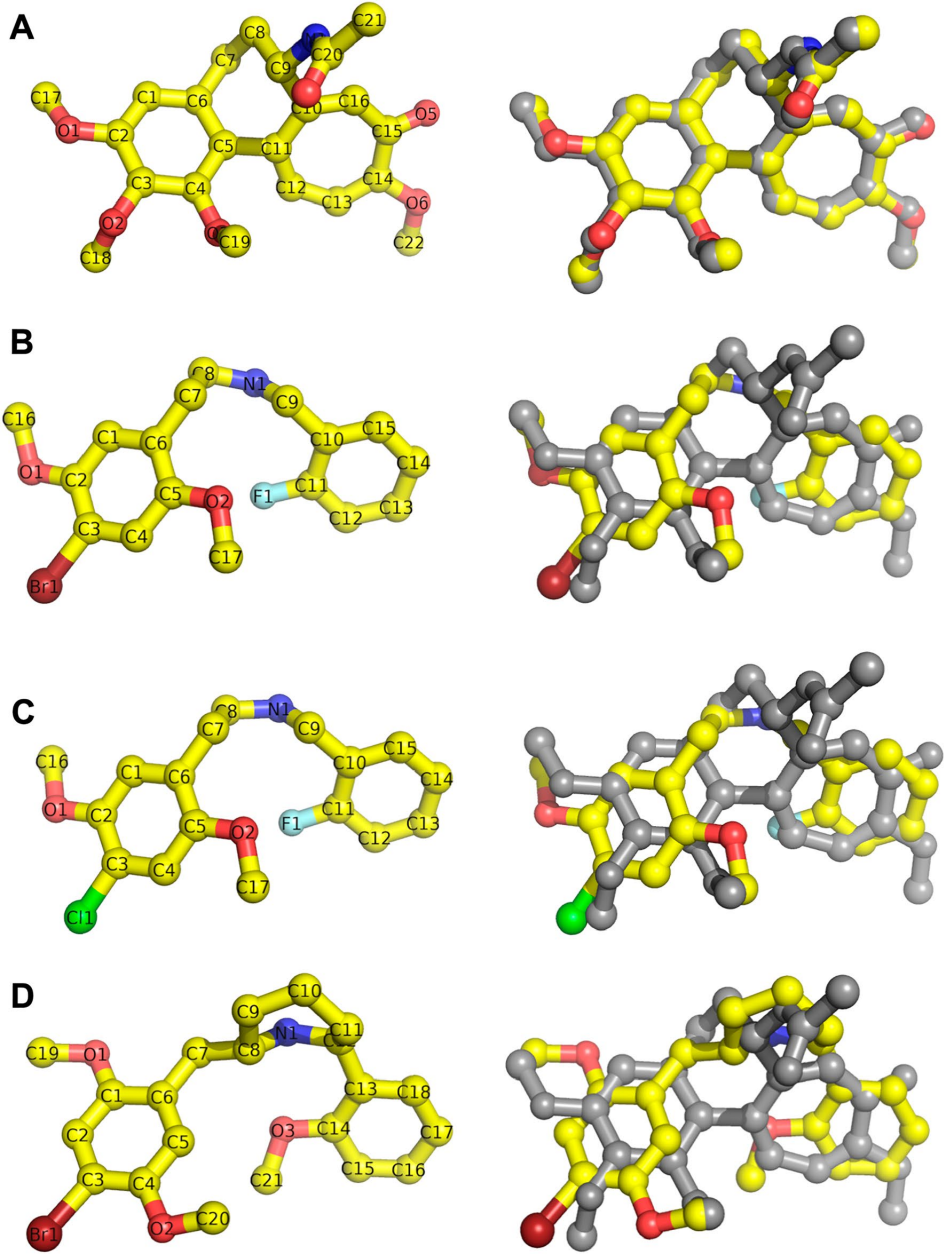
A stick and ball representation using Pymol of docked colchicine, 25B-NBF, and 25C-NBF, DMBMPP and the crystal structure of colchicine. The docked structure of colchicine, 25B-NBF, 25C-NBF and DMBMPP follows this atomic coloring scheme: C: yellow, O: red, N: blue, Cl: green, Br: maroon, FL: light blue. (**A**) Colchicine (yellow) in a docked pose (left), and the overlay of the crystal pose of colchicine (grey) from PDB 4O2B and the docked colchicine (yellow)(right), RMSD = 0.2593 Å. (**B**) The docked pose of 25B-NBF (yellow) (left), and the overlay of the crystal pose of colchicine (grey) and the docked 25B-NBF (yellow)(right). (**C**) The docked pose of 25C-NBF (yellow) (left), and the overlay of the crystal pose of colchicine (grey) and the docked 25C-NBF (yellow) (right). (**D**) The docked pose of DMBMPP (yellow) (left), and the overlay of the crystal pose of colchicine (grey) and the docked DMBMPP (yellow) (right).

To validate the accuracy of our docking protocol parameters we calculated the RMSD of the crystal and docked poses of colchicine. The RMSD of 0.2593 Å is well below the 2.3 Å resolution of the 4O2B PDB crystal structure confirming the ability of the chosen parameters to accurately predict the crystallographic binding pose of colchicine (Fig. 2A).

As two of the methoxy groups on colchicine’s trimethoxy benzene ring are important for binding and activity (i.e. C1-methoxy improves activity and coordinates the correct binding conformation, C5-methoxy is essential for binding, according to the Fig. 2A conventions) the binding poses of 25B-NBF, 25C-NBF, and DMBMPP were compared to amino acid residues within 5 Å of these methoxy groups^26^ (Fig. 2, Table 2).

**Table 2:** β-tubulin residues interacting with colchicine, 25B-NBF, 25C-NBF, and DMBMPP.

Ligand	Group	β-tubulin residues
Colchicine	C4-methoxy	**C241, L248, A316, A317, K352, T353, A354**
	C2-methoxy	**C241, L242, L248, A250, D251, L255**
25B-NBF	C5-methoxy	**C241, L248, A316, A317, K352, T353, A354**
	C2-methoxy	**C241, L242, A250, D251**, L252, **L255**, N258, M259, V315, A316, **K352**
25C-NBF	C5-methoxy	**C241,** L242, **L248,** A250, D251, L252, L255, N258, **A316, A317**, **K352, T353, A354**
	C2-methoxy	**C241, L242, L248, A250, D251**, L252, **L255**, A316, A317, I318, K352, T353, A354
DMBMPP	C4-methoxy	**C241, L248**, A250, **A317**, I318, **K352, T353, A354**
	C1-methoxy	**C241, L242, A250, D251,** L252,** L255**

Bold indicates residues identified as key to colchicine binding.

### In vitro microtubule polymerization assays

**Figure 3:**
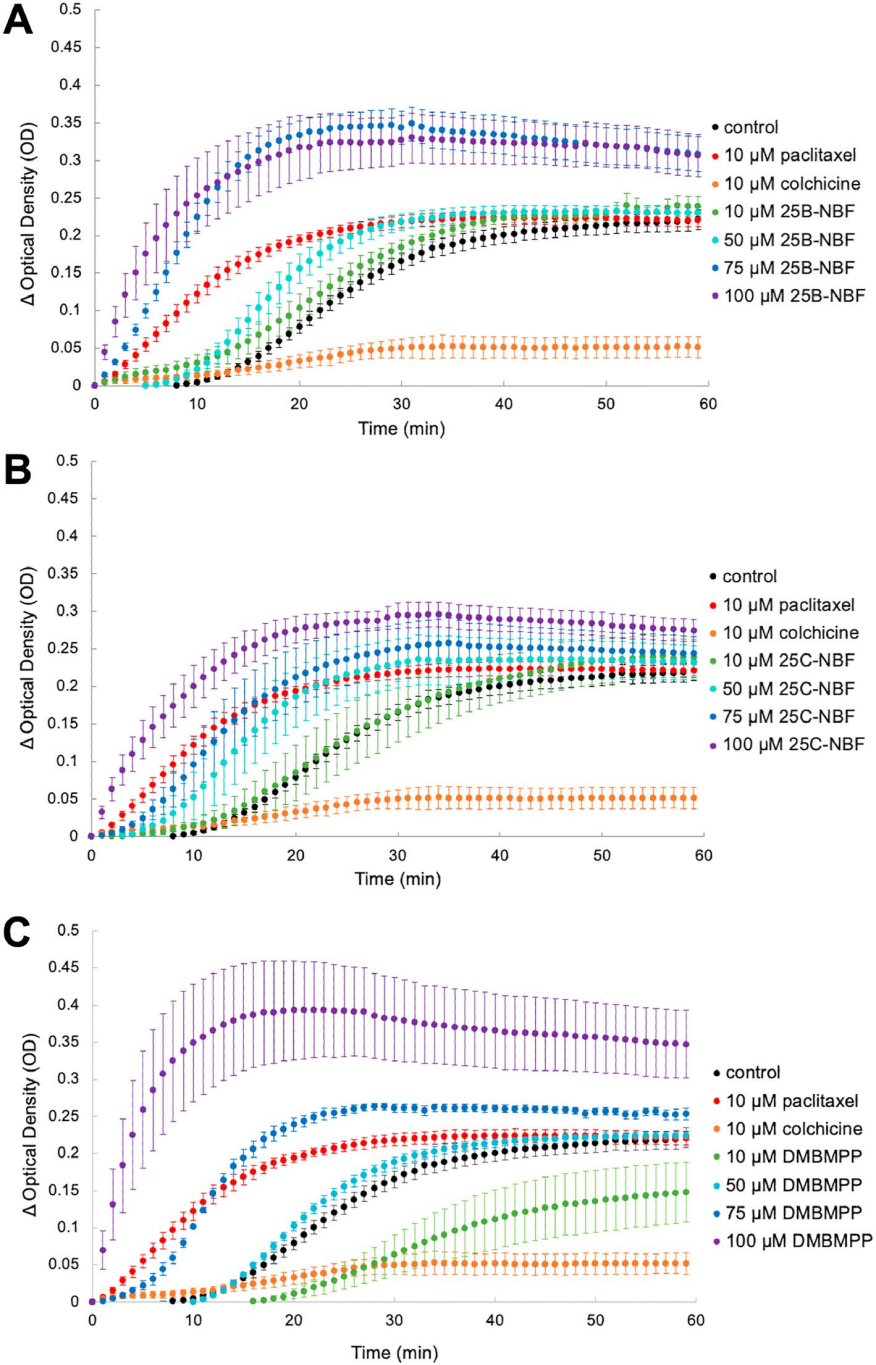
Polymerization rate curve of change in optical density at 355 nm over time of (**A**) 25B-NBF at 10 µM (green), 50 µM (cyan), 75 µM (blue), 100 µM (purple) compared to control (black), positive control 10 µM paclitaxel (red), and negative control 10 µM colchicine (orange); (**B**) 25C-NBF at 10 µM (green), 50 µM (cyan), 75 µM (blue), 100 µM (purple) compared to control (black), positive control 10 µM paclitaxel (red), and negative control 10 µM colchicine (orange); (**C**) DMBMPP at 75 µM (blue), 100 µM (purple) compared to control (black), positive control 10 µM paclitaxel (red), and negative control 10 µM colchicine (orange). Averages plotted from a minimum of three biological replicates. Error bars are standard error of the mean.

Tubulin polymerization assays were measured via optical density at 355 nm. Normalized data for all replicates may be found in Supplementary Information Table S2. As compared to control, the rate of polymerization of tubulin increased as the concentration of 25B-NBF and 25C-NBF increased from (10–100 µM) (Fig. 3A; Table 3). In the presence of 50 µM, 75 µM, and 100 µM 25B-NBF tubulin’s polymerization rate increased significantly above control (*p* = 0.002, 0.0001, and 0.01, respectively) while 10 µM 25B-NBF showed no appreciable change compared to the control (*p* = 0.598) (Fig. 3A; Table 3). At concentrations of 75 µM 25B-NBF, the microtubule polymerization rate was significantly higher than polymerization with paclitaxel (10 µM) a known potent microtubule stabilizing agent (*p* = 0.010). While at 100 µM 25B-NBF this trend remained, it was not at statistical significance owing to the larger variance in this polymerization condition (*p* = 0.058).

**Table 3:** T-test statistical comparison of peak tubulin polymerization rates and times to half maximum for experimental conditions versus control (Mean ± SEM).

Chemical dose	Rate (mOD/min)	*p*
Control	11.0 ± 0.2	–
Paclitaxel 10 µM	15.1 ± 0.8	0.020
Colchicine 10 µM	4.8 ± 1.2	0.006
25B-NBF 10 µM	11.5 ± 0.3	0.598
25B-NBF 50 µM	17.0 ± 0.7	0.002
25B-NBF 75 µM	27.1 ± 1.1	< 0.001
25B-NBF 100 µM	45.5 ± 4.5	0.010
25C-NBF 10 µM	11.7 ± 1.7	0.764
25C-NBF 50 µM	19.8 ± 1.2	0.006
25C-NBF 75 µM	19.7 ± 2.1	0.073
25C-NBF 100 µM	34.2 ± 3.7	0.013
DMBMPP 10 µM	6.7 ± 1.5	0.061
DMBMPP 50 µM	13.5 ± 0.4	0.036
DMBMPP 75 µM	22.7 ± 2.9	0.010
DMBMPP 100 µM	75.3 ± 11.0	0.038

The effect of 25C-NBF on tubulin was different from 25B-NBF despite their structure being almost identical except for change of bromine to chlorine at C4 (Fig. 2B,C). Both 50 µM and 100 µM 25C-NBF showed a significant increase in the polymerization rate compared to control (*p* = 0.006 and 0.013, respectively) (Fig. 3B). The 75 µM 25C-NBF condition trended with a higher polymerization rate compared to control, however, again this was not significant due to the larger variance in this condition (*p* = 0.073). Unlike 25B-NBF, higher concentrations of 25C-NBF did not show a significant increase in polymerization over paclitaxel, although the polymerization rate at 100 µM of 25C-NBF trended higher than paclitaxel *p* = 0.087, again with no significance due to the high variance in this measure.

In the presence of 10 µM DMBMPP there was a trending decrease in the rate of tubulin polymerization compared to the control condition (*p* = 0.061) which showed a comparable rate to colchicine (*p* = 0.6183) (Fig. 3C). However, at a concentration of 50 µM DMBMPP the polymerization rate was significantly higher than control (*p* = 0.036). Concentrations of 75 and 100 µM DMBMPP enhanced polymerization significantly over control (*p* = 0.010 and 0.038, respectively). While the mean rate of 75 and 100 µM DMBMPP was found to be much higher than paclitaxel, this difference was not found to be significant owing to the large variance in these measures, particularly for 100 µM.

### Microtubule staining and imaging

As we did not know if the change in tubulin polymerization rate was mediated by interaction with 25B-NBF, 25C-NBF, and DMBMPP or if concentrations of the compounds led to aberrant aggregate forms of tubulin to alter the optical density reading, we conducted in vitro polymerization experiments with fluorescent tagged microtubule protein.

**Figure 4:**
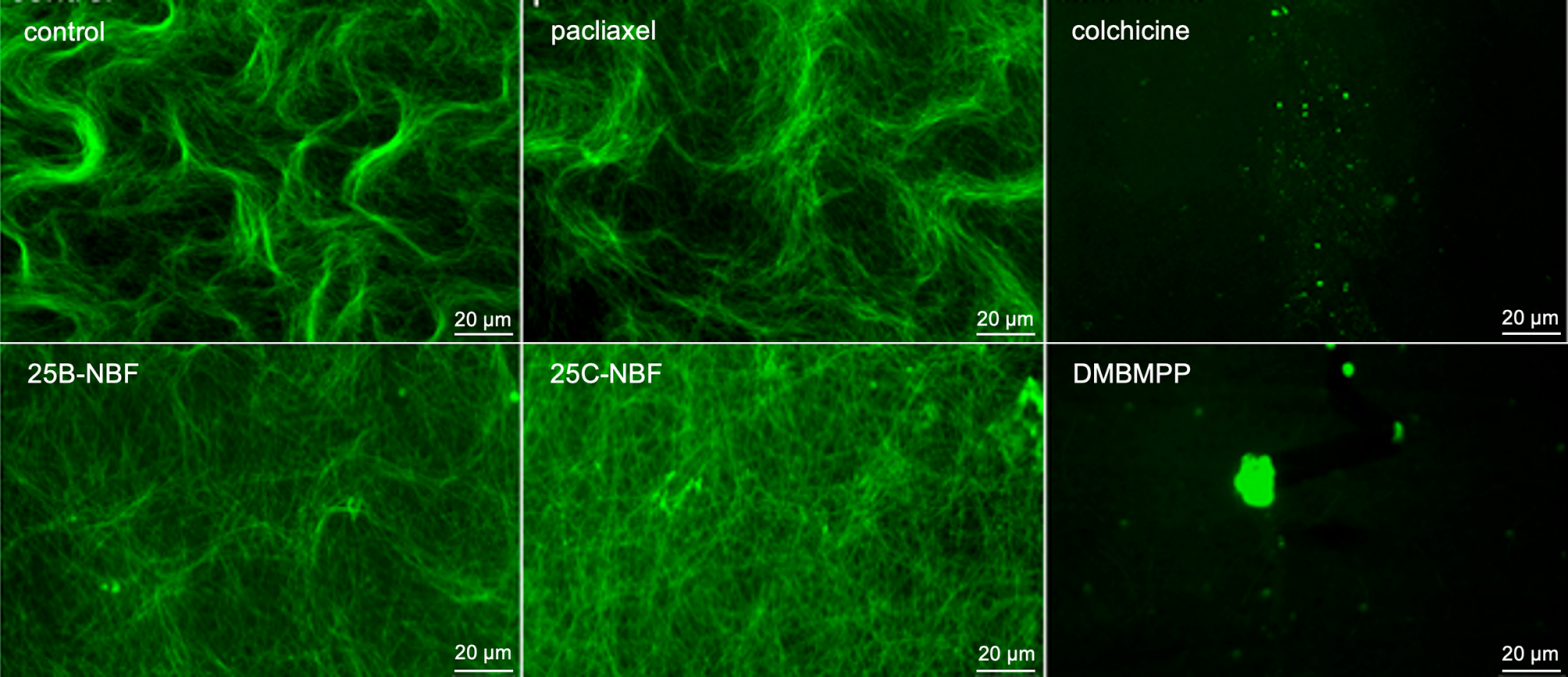
Fluorescent imaging of control, 10 µM paclitaxel, 10 µM colchicine, 100 µM 25B-NBF, 100 µM 25C-NBF and 100 µM DMBMPP after in vitro polymerization assay at a magnification of 50.4×.

As shown in Fig. 4, microscope images revealed the presence of 25 nm wide filaments in the control and 10 µM paclitaxel conditions indicating microtubule polymerization as expected. Due to the protein concentration used bundled forms are observed. The presence of 10 µM colchicine led to small aggregates of tubulin and a lack of filamentous structures indicating no microtubule polymerization also as expected. In the presence of 100 µM 25B-NBF and 25C-NBF 25 nm wide filaments were also observed indicating the presence of polymerized microtubules. The lack of bundling observed in these conditions is attributed to a high degree of unbound compound in solution. Finally, 100 µM DMBMPP resulted in large aggregate forms of various size and shape, indicating that DMBMPP inhibits proper microtubule polymerization. This is part explains the large variation for the 100 µM DMBMPP in the optical density measures observed in the polymerization assays, owing to the variable size of the resulting aggregates during each replicate.

## Discussion

Here we examined the effects of phenethylamine designer psychedelic drugs on microtubule polymerization dynamics. Computational docking analysis predicted that the 110 substituted phenethylamines investigated possessed moderate to strong binding affinity for the tubulin protein in the colchicine binding pocket with the top 10% showing comparable, but slightly less affinity that colchicine itself. Based on predicted absorption, distribution, metabolism, and excretion pharmacokinetic and physicochemical properties the effect on microtubule polymerization dynamics of three of these top compounds were investigated. It was found that all three substituted phenethylamines resulted in enhancing the rate of microtubule polymerization in a concentration dependent manner similar to previous findings with 2-phenethylamine^10–12^. This suggests a biochemical basis for neuromodulation via direct effect on tubulin of the phenethylamines 25B-NBF, 25C-NBF and DMBMPP that may contribute to (1) their adverse effects, (2) their main psychedelic effects, and/or (3) may be harnessed for use in the treatment of cancers, neurodegenerative disease, and neuropsychological illnesses.

While our initial computational screening indicates that the phenethylamine compounds investigated bind to the colchicine binding site with a moderate to strong strength, it is still unclear if this is the site of action contributing to the observed enhancement of microtubule polymerization. The colchicine binding site is most commonly targeted as a site for microtubule polymerization inhibition^27,28^. As such, the seemingly enhanced polymerization rates observed in the in vitro polymerization assays with 25B-NBF and 25C-NBF was unexpected. However, noscapine, a benzylisoquinoline alkaloid isolated from poppy extract, which is structurally similar to colchicine in that both contain a dimethoxy phenyl group, is predicted to bind to the colchicine binding site and is also known to stabilize MT leading to their polymerization at a lesser extent compared to paclitaxel^29,30^. This increase in polymerization has been ascribed to reducing the dynamic instability of microtubules by increasing the stopping time of microtubules^31^. Our results here are consistent with a similar mechanism.

Phenethylamines, including 25B-NBF, 25C-NBF, and DMBMPP, act as potent agonists and partial agonists for human 5-HT_2A_ receptors^32^. While this activity is traditionally viewed as originating at the plasma membrane, there is increasing evidence that suggests that G-protein coupled receptor signaling from intracellular compartments plays an important roles in the cellular response to drugs^33^. Specifically, Vargas et al. recently demonstrated that intracellular 5-HT_2A_ receptors mediate the plasticity-promoting properties of the membrane-permeable tryptamine psychedelics DMT (*N*,*N*-dimethyltryptamine) and psilocybin, but not chemically modified versions unable to cross the membrane^33^. While it remains to be seen if the same occurs for the phenethylamine psychedelics, it does highlight the importance of the intracellular location of action. This is of key relevance to our findings here as the 5-HT_2A_ receptor and the light chain 2 domain of the microtubule-associated protein MAP1A are co-localized in the intracellular compartment of pyramidal neuronal dendrites of adult rats, suggesting the association of 5-HT_2A_ receptors with the cytoskeleton in cortical neurons in vivo^34^. Additionally, activation of 5-HT_2A_ receptors by hallucinogens significantly attenuates the effect of the 5-HT_1A_ receptor on NMDA receptor currents and microtubule depolymerization in frontal cortex pyramidal neurons suggesting that intraneuronal 5-HT receptor signaling processes involve cytoskeletal elements^35^. Finally, depolymerization of microtubules prolongs the desensitization of 5-HT receptors, suggesting a functional relationship between 5-HT receptors and the microtubule cytoskeleton^36^. Overall, this suggests a mechanism by which direct action of phenethylamines on microtubule dynamics may affect the cellular response to psychedelic phenethylamines.

A body of work has been done on how to identify the presence of these drugs (25B-NBF, 25C-NBF, and DMBMPP) in the human body for drug detection purposes^37–41^, however little is known about their overall effects and mechanisms of action including potential effects on the microtubule cytoskeleton. As such, there is very limited dosage research for 25B-NBF, 25C-NBF, and DMBMPP. This is in part due to the illicit use of phenethylamines. Even the well-known research work of the Shulgins^42^, which describes in detail phenethylamines and their physical properties, personal dosages used, duration of effects observed, and commentary on effects, does not contain information on these compounds. However, all three of the compounds studied here experimentally, 25B-NBF, 25C-NBF, and DMBMPP, are derivatives of the phenethylamine psychedelic 2C-B, which is indicated to have psychedelic effect at dosages between 12 and 24 mg^42^. Even so, this is not easily translatable to the concentrations used in our experiments due to the need to account for the mode of administration on distribution throughout the body. As a naïve estimate using the molecular weights of 25B-NBF, 25C-NBF, and DMBMPP (360, 405, and 420 g/mol, respectively) and a weight of 12–24 mg yields concentrations ranging between 30 and 67 µM comparable to the range we see effect on tubulin polymerization in our experiments. This suggests that recreational usage doses may have immediate effect on the microtubule cytoskeleton.

Very recent work indicates that 25C-NBF has addictive and neurotoxic properties as determined by deficits in motor coordination and memory in a murine model^43^, however there has been increasing association of the potential for phenethylamines in the treatment of illness. Specifically, the discovery of trace amine-associated receptor 1 (TAAR1), which modulates dopamine transmission, marks a target for phenthylamines to exert direct control over dopaminergic neuron firing and release having implications for both the pathophysiology of, and treatment design for, disorders that involving aminergic dysregulation such as Parkinson's disease, schizophrenia, mood disorders, and addiction^44^. Phenethylamines have been shown to readily cross the blood–brain barrier in a rodent model^45^, consistent with our calculations here. This ability to quickly cross the blood brain barrier and alter microtubule dynamics marks phenethylamines as potential psychoplastogens; fast-acting therapeutics, capable of rapidly promoting structural and functional neural plasticity^46^. This also means that they have potential for detrimental effects if not used in a controlled manner. Clearly further investigations into these mechanisms are warranted.

## Methods

### Protein preparation

The Protein Databank^47^ structure PDB 4O2B was used to model the tubulin-colchicine complex. One tubulin dimer was taken from this structure and prepared using the Protein Preparation Prepwizard tool^48^ in Schrodinger Suites version 2016-2. We adjusted bonds, added hydrogens, and relaxed the protein structure into a more energetically favorable confirmation. As none of the water molecules within 1 nm of colchicine in the 4O2B structure were found to form any hydrogen bond bridging between colchicine and tubulin protein amino acids we removed all waters from the protein. After relaxation, the colchicine binding site was identified, and the colchicine molecule was removed from the tubulin structure. The tubulin protein structure was then prepared for docking using AutodockTools 1.5.68^49^ by removing nonpolar hydrogens and adding Kolman United Atom Charges.

### Ligand preparation

Structures for 110 psychedelic phenethylamine ligands identified from an online list^50^ were acquired from ChemSpider^51^ or PubChem^52^ databases as either SDF or mol files. They were converted into PDB structure files through the NIH Online SMILES Translator and Structure File Generator (https://cactus.nci.nih.gov/translate/). The Ligand Preparation tool^53^ in Schrodinger Suites version 2016-2 was utilized to add hydrogens, neutralize charged groups, and enumerate tautomer and protonation states using Epik with the Hammett and Taft methodology^54^. The most probable tautomer was selected for use in Autodock Vina for efficiency as using the most probable tautomer has been shown to be better than docking the entire enumeration ensemble, as the scoring functions are generally not accurate enough to discriminate among them^55^. The ligands were then prepared for docking in Autodock Vina using AutoDockTools 1.5.68 to add rotatable bonds and Gasteiger Charges to ensure proper parameterization for docking.

### Computational docking

We used computational docking to predict the conformation and binding energy of colchicine and 110 phenethylamine compounds in the binding site of colchicine on a crystal structure of tubulin (PDB ID: 4O2B). This was done with AutoDock Vina^21^ with exhaustiveness of 300 in a 20 Å cubed search space at the colchicine binding site and a resolution of 0.994 Å. The coordinates for the grid box centered on the colchicine binding site are 14.694 for x, 6.278 for y, and − 19.028 for z dimensions. To verify protocol accuracy, the root mean squared deviation (RMSD) between the docked and crystallographic pose was calculated and found to be less than 0.3 Å. Docked phenethylamine compounds were also compared to the crystal binding pose of colchicine via RMSD. RMSD was calculated using the RMSD tool in Visual Molecular Dynamics (VMD)^56^. The lowest energy binding pose (LE) was used to rank the docked compounds.

### LightBBB

We measured the ability of colchicine and each phenylethylamine compound to cross the BBB by using LightBBB^23^. LightBBB makes predictions on the BBB permeability of compounds through the database of over 7000 compounds from the SMILES repository, each one with classified BBB permeability. The model was trained using the Light Gradient Boosting Machine algorithm to make the necessary predictions with an accuracy of 89%, specificity of 0.77, and sensitivity of 0.93.

### SwissADME

SwissADME evaluates the ADME (Absorption, Distribution, Metabolism, and Excretion) pharmacokinetic and physicochemical properties of the drugs^22^. It was used to evalute the number of pan-assay interference compounds (PAINS) alerts and the number of Brenk alerts for each of the 110 phenethylamine compounds, and whether the compounds act as P-glycoprotein (Pg-p) substrates, or inhibitors of five enzymes from the Cytochromes P450 (CYP) family.

### In vitro tubulin polymerization assay

The tubulin polymerization Assay (BK006P) was purchased from Cytoskeleton Inc. (Denver, CO, USA) and it was used with the purpose to measure the optical density of each sample over time using a spectrophotometer at a temperature of 37 °C at a wavelength of 355 nm on 96 well-plates. The tubulin protein was purified from porcine brain and diluted in General Tubulin Buffer (GTB), the final reaction concentration of tubulin was 3 mg/ml. Paclitaxel (Cytoskeleton Inc.) and colchicine (C9754, Sigma, St. Louis, MO, USA) were resuspended in DMSO and diluted in GTB for a final compound concentration of 10 µM. 25B-NBF (15967, Cayman Chemical, Ann Arbor, MI, USA), 25C-NBF (15969, Caymen Chemical, Ann Arbor, MI, USA), and DMPMPP (GLXC-22812, Glixx Labs, Hopkinton, MA, USA) were diluted in DMSO for a stock of 2 mM and then further diluted in GTB for different final concentrations of 10 µM, 50 µM, 75 µM and 100 µM. The final concentration of the polymerization reaction was 80 mM PIPES, 2.0 mM MgCl_2_, 0.5 mM EGTA, 60% v/v glycerol, pH of 6.9, and 10 µM of guanosine triphosphate (GTP). Polymerization was measured every minute in a Victor Nivo (Perkin Elmer, Waltham, MA) at an optical density (OD) of 355 nm. The microplate was continuously disturbed using the orbital shaking function of the plate reader in between measurements as recommended by the manufacturer.

### Statistics

The maximum rate of polymerization was obtained from each replicate for each condition by finding the maximum gradient of the polymerization curve using the *gradient* function in MATLAB over one-minute intervals. Means and standard error of the mean (SEM) were calculated for each condition from these distributions. Two-tailed t-tests were used to compare drug conditions. Comparisons with p-values less than 0.050 were taken as significantly different, while comparisons with p-values less than 0.100 were taken to show a trend.

### Microtubule staining and imaging

We used the HiLyte Fluor 488 labelled kit (TL488M) purchased from Cytoskeleton Inc. (Denver, CO, USA). The tubulin protein of TL488M was purified from the porcine brain, and it was covalently linked to HiLyte Fluor 488 at random surface lysines. We mixed tubulin from the TL488 kit and tubulin from the BK006P in a ratio of 1:9, yielding a final tubulin concentration of 3 mg/ml for the polymerization assay. We performed the same protocol of the polymerization assay as mentioned in the methods section, except for using a 96-well plate we used 1.5 ml tubes for each reaction that were previously incubated 1 h prior to the assay at 37 °C, and then the tubes were incubated again for 1 h at 37 °C during the assay. The final reaction concentration of the buffer was 80 mM Piperazine-*N,N'*-bis[2- ethanesulfonic acid] sequisodium salt, 2.0 mM Magnesium chloride, 0.5 mM Ethylene glycol-*bis*(b-amino-ethyl ether) *N,N,N',N'*-tetra-acetic acid, 60% v/v glycerol, pH of 6.9, and 10 µM of guanosine triphosphate (GTP). The final concentration of colchicine and paclitaxel was 10 µM and the final concentration of 25B-NBF, 25C-NBF was 100 µM. After the incubation of 1 h was over, we mounted each reaction in a slide and placed a cover slip on it. We used the GFP filter of an IX73P2F Fluorescent Microscope from Olympus (Center Valley, PA) to visualize the samples at a magnification of 50.4x.

### Supplementary Information

Supplementary Table 1.

Supplementary Table 2.

## Data Availability

The datasets generated during and/or analyzed during the current study are available in the supplementary information.
